# Effect of Zinc Supplementation on the Serum Metabolites Profile at the Early Stage of Breast Cancer in Rats

**DOI:** 10.3390/nu12113457

**Published:** 2020-11-11

**Authors:** Barbara Bobrowska-Korczak, Paulina Gątarek, Dorota Skrajnowska, Wojciech Bielecki, Rafal Wyrebiak, Tomas Kovalczuk, Robert Wrzesień, Joanna Kałużna-Czaplińska

**Affiliations:** 1 Department of Bromatology, Faculty of Pharmacy with the Laboratory Medicine Division, Medical University of Warsaw, Stefana Banacha 1, 02-097 Warsaw, Poland; dorota.skrajnowska@wum.edu.pl; 2 Department of Chemistry, Institute of General and Ecological Chemistry, Lodz University of Technology, Stefana Zeromskiego 116, 90-924 Lodz, Poland; gatarekpaulina@gmail.com (P.G.); joanna.kaluzna-czaplinska@p.lodz.pl (J.K.-C.); 3 Department of Pathology and Veterinary Diagnostics, Faculty of Veterinary Medicine, Warsaw University of Live Sciences, Nowoursynowska 159c, 02-787 Warsaw, Poland; wojciech_bielecki@sggw.pl; 4 Department of Biomaterials Chemistry, Analytical Chemistry and Biomaterials, Faculty of Pharmacy with the Laboratory Medicine Division, Medical University of Warsaw, Stefana Banacha 1, 02-097 Warsaw, Poland; rafal.wyrebiak@gmail.com; 5 LECO Instrumente Plzen, Plaska 66, 323 00 Plzen, Czech Republic; tomas.kovalczuk@leco.cz; 6 Central Laboratory of Experimental Animals, Medical University of Warsaw, Stefana Banacha 1a, 02-091 Warsaw, Poland; robert.wrzesien@wum.edu.pl

**Keywords:** breast cancer, zinc nanoparticles, metabolomic

## Abstract

The cytotoxic properties of zinc nanoparticles have been evaluated in vitro against several types of cancer. However, there is a lack of significant evidence of their activity in vivo, and a potential therapeutic application remains limited. Herein we report the effective inhibition of tumor growth by zinc nanoparticles in vivo, as the effect of the dietary intervention, after the chemical induction in a rodent model of breast cancer. Biopsy images indicated grade 1 tumors with multiple inflammatory infiltrates in the group treated with zinc nanoparticles, whereas, in the other groups, a moderately differentiated grade 2 *adenocarcinoma* was identified. Moreover, after the supplementation with zinc nanoparticles, the levels of several metabolites associated with cancer metabolism, important to its survival, were found to have been altered. We also revealed that the biological activity of zinc in vivo depends on the size of applied particles, as the treatment with zinc microparticles has not had much effect on cancer progression.

## 1. Introduction

As the World Research Cancer Fund (WCRF) reports, breast cancer is one of the most common cancer malignancies in women worldwide and the second cause of cancer death overall. In 2018 over 2 million new cases were recorded, the highest rate of which occurred in developed countries [1]. Current clinical approaches to breast cancer are considered to be invasive, of low-specificity and may generate severe side effects [2]. Therefore, a potential chance arises for nanotechnology-derived solutions. The nanoparticles (NP) overcome biological barriers readily and enable prolonged blood circulation time, which, in effect, enhances the accumulation of nanoparticles in cancer cells [2]. Zinc, particularly in the form of nanoparticles, is a promising target for nanomedicine applications in view of its biological significance and strong safety record [3,4,5,6,7].

To date, a vast majority of studies on Zn NP toxicity toward cancer have been done *in vitro*. For instance, the activity of Zn NP was demonstrated against leukemias, lymphomas, glioma, as well as breast, bone, colon [4], liver [8,9], cervical [10] and ovarian cancer [11]. In all of the above-mentioned cases, an inhibitory impact on the proliferation and viability of cancer cells was observed [4,9,10,11]. However, the mechanics of Zn NP cytotoxicity has not been completely understood [4,5,12]. The oxidative stress-based apoptosis has been widely deliberated, as Zn NP may generate reactive oxygen species (ROS), similar to other metals/metal oxides nanoparticles. Excessive ROS levels cause irreparable damage in the cell, leading to its apoptosis [4,7]. Kadhem et al. and Moghaddam et al. observed this effect in MCF-7 cells treated with ZnO NP. [13,14]. In these models, pro-apoptotic *p53* [13,14], *p21, Bax, JNK* [14] and *CAS* [13] genes were upregulated, whereas anti-apoptotic genes *bcl-2, AKT1* and *ERK1/2* were downregulated [14]. Moreover, the Zn NP toxic effect was found to be selective toward fast proliferating cells [12,15]. For example, Akhtar et al. observed the Zn NP-mediated apoptosis in cancer cells via the p53 pathway had no impact on normal rat astrocytes and hepatocytes under the same treatment conditions [12].

Unfortunately, the activity of Zn NP in vivo has not been investigated in depth, although a wide range of cancer cell lines responded to the Zn NP treatment in vitro. The cytotoxicity of ZnO NP against cancer was demonstrated for the first time in 2016 by Hassan et al. in the case of chemically induced human hepatocellular carcinoma (HCC) in rats [9]. Furthermore, the anti-cancer effect of biosynthesized Zn NP was also found by Raajshree et al. in the murine model of Dalton’s lymphoma ascites [16]. Tanino et al. reported genotoxicity of ZnO NP and low viability of small-lung cancer in mice in response to treatment [17]. In most cases, effects on normal cells were minimal, and effective tumor inhibition was found.

To date, no evaluation of therapeutic response to Zn NP in breast cancer in vivo has been performed, although multiple reports indicate a significant impact on MCF-7 cell lines proliferation and viability. Therefore, in this paper, we investigate the cytotoxicity of zinc micro- and nanoparticles to chemically induced breast *adenocarcinoma*, based on a rodent model. We used GC/TOFMS profiling to comprehensively identify metabolic alterations in response to treatment. Our findings may contribute to the discussion on low-invasive therapeutic strategies against breast cancer.

## 2. Materials and Methods

### 2.1. Preparation of Zinc Micro- and Nanoparticles

**Figure 1:**
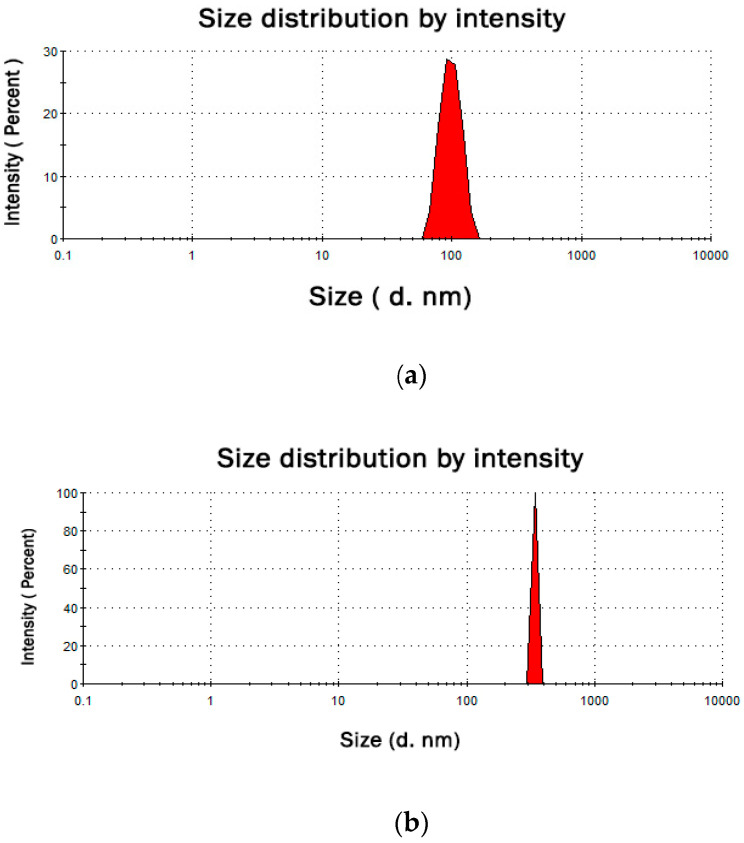
DLS size distribution graph for synthesized zinc nano- (**a**) and microparticles (**b**).

The synthesis of micro- and nanoparticles of zinc was performed following the previously published protocol by Ghant et al. [18]. 1.36 g of anhydrous zinc chloride (analytical grade, Ubichem Limited, Worcestershire, UK) and 50 mL of dry 1,3,5–trimethylbenzene (97%+, TCI EUROPE N.V., Zwijndrecht, Belgium) were added to a 100 mL round bottomed flask with Dean–Stark receiver. The mixture was stirred and heated until 15 mL of solvent were distilled off. Next, 330 mg of PVP (polyvinylpyrrolidone K15, Mw 10,000, TCI EUROPE N.V., Zwijndrecht, Belgium) and 15 mL of 1,3,5–trimethylbenzene were added, and another 15 mL of solvent was distilled off to remove water traces. Upon the reaction mixture was cooled to 0 °C, LiAlH4 (760 mg) (powder, 95%+, TCI EUROPE N.V., Zwijndrecht, Belgium) was added, and the Dean–Stark apparatus was replaced with a reflux condenser. Further, for the zinc nanoparticles preparation, the reaction mixture was refluxed and vigorously stirred for 24 h, whereas zinc microparticles were obtained by heating the reaction mixture to 100 °C and vigorously stirred for 24 h. Finally, both reaction mixtures were cooled down to ambient temperature, and cold methanol was slowly poured to decompose the unreacted LiAlH4. The obtained products were centrifuged at 3500 rpm for 6 min, washed several times with cold methanol and subsequently dried. The synthesized zinc micro- and nanoparticles were stabilized with PVP to prevent oxidation and aggregation. In order to examine the average size of particles and their zeta potential, the dynamic light scattering technique (DLS) was applied. The Zetasizer Nano ZS instrument (Malvern Instruments, Westborough, MA, USA) equipped with a red laser with a wavelength of 633 nm and a scattering angle of 173° at 25 °C was utilized for this purpose (Table 1, Figure 1).

### 2.2. Rodent Model

Twenty-four female Sprague–Dawley rats were obtained from the Animal Laboratory of the Department of General and Experimental Pathology, Medical University of Warsaw. All animals were fed a standard diet (Labofeed H, Kcynia, Poland) and water ad libitum. Rats were maintained in a 12:12-h light–dark cycle at a constant temperature of 22 °C. The animals were assigned to three experimental groups: (i) rats, which were fed only the standard diet—without supplementation, receiving 0.4 mL of water—s, (ii) rats with the microsized zinc particles (342 nm) supplementation at a dose of 4.6 mg/mL, administered in 0.4 mL of water, orally, via *gavage*—m, (iii) rats with the nanosized zinc particles (99 nm) supplementation, administered orally, via *gavage*, at the same dose of 4.6 mg/mL in 0.4 mL of water—n. The zinc supplementation was included in the rats’ diet from 40 days until 20 weeks of age, and the dose exceeded the standard fodder zinc content two times, i.e., 76.9 mg/kg diet. To induce mammary cancer (*adenocarcinoma*), rats were treated twice via *gavage* with DMBA dissolved in rapeseed oil (7,12-dimethyl-1,2-benz[a]anthracene; Sigma-Aldrich, St. Louis, MO, USA). The first treatment was performed at 60 days of age (80 mg/kg of body weight), followed by another dose of 40 mg/kg of body weight at 90 days of age. To indicate the development of tumors, animals were examined by palpation during the course of the study. Figure S1 presents a schematic representation of the design of the study.

All of the procedures involving animal experimentation were approved by the Animal Ethics Committee of the Faculty of Biology, University of Warsaw (no. of approval 645/2018 issued on 3 July 2018).

### 2.3. Preparation of Biological Material and Histopathological Examination

The animals were sacrificed by decapitation at 150 days of age in order to histopathologically examine tumors and to perform a comprehensive GC/MS screening of the blood serum metabolome. The survival rates of the test groups were 100%. The obtained blood serum was aliquoted to avoid freezing-thawing cycles and stored at −80 °C for further analyses. The tissues were fixed in a buffered formalin solution, then dehydrated, sealed in paraffin and cut into 4 µm sections. Hematoxylin and eosin staining of tissue sections was performed, and samples were imaged using a BX43 Olympus microscope.

### 2.4. Solvents and Derivatizing Agents for GC/MS Analysis

Acetonitrile, isopropanol, water, methanol and pyridine, of LC or GC grade were provided by Merck Darmstadt, Germany. Both derivatizing agents, i.e., methoxyamine hydrochloride and N-methyl-N-(trimethylsilyl) trifluoroacetamide (MSTFA), were purchased in Sigma Aldrich St. Louis, MO, USA (purity > 98%).

### 2.5. Extraction of Metabolites from Blood Serum and Derivatization

Extraction of metabolites was performed according to the method proposed recently by Fiehn [19]. Blood serum samples were transported on dry ice to avoid uncontrolled thawing, and then samples were kept on ice, chilled at 0 °C to defrost slowly. To ensure homogenization, samples were carefully vortexed for 10 s and shortly centrifuged before 30 µL aliquot of serum was taken. For the extraction, 1 mL of an ice-cold mixture of acetonitrile, isopropanol and water (3:3:2 v/v/v) was used. Samples were vortexed for about 10 s, then shaken at 4 °C for 5 min and finally centrifuged for 2 min at 13,000 RCF (4 °C). Supernatants were removed and split into two 450 µL portions—first, one for a backup sample, and the second one to be subjected to analysis. Extraction solvents were evaporated at 30 °C in a rotary vacuum concentrator (Eppendorf). All samples with repetitions, biological pools, QCs as well as reagent and solvent blanks were prepared and derivatized as one complete set. For the derivatization, 10 µL of methoxyamine hydrochloride solution (20 mg/mL in dry pyridine) were added and samples were kept at 37 °C for 90 min with agitation (900 rpm). Next, silylation was done with 90 µL of MSTFA, at 37 °C, for 30 min with constant agitation (900 rpm). The whole set was then centrifuged at room temperature, transferred to vials and immediately subjected to GC/MS analysis.

### 2.6. GC/MS System and Analysis Method

To determine components of blood serum metabolome, the GC/MS system was used, consisting of the Agilent 7890B gas chromatograph with S/SL inlet (Agilent Technologies) connected to the Pegasus BT time-of-flight mass spectrometer (LECO Corporation). The Rxi-5MS fused-silica capillary column of low-polarity bonded-phase was applied for separation (30 m length, 0.25 mm ID and 0.25 μm film thickness) (Restek Bellefonte, PA, USA). The constant flow of 1 mL/min was set, and helium was used as a carrier gas. Each sample in the amount of 0.5 µL was injected in the splitless mode at 280 °C. The inlet purge flow rate was set to 40 mL/min and was turned on 70 s after injection. In addition, the septum was purged with 3 mL/min, respectively. The following GC oven temperature program was selected: 1 min at 70 °C, raised subsequently by 12 °C/min to 300 °C and held for 14 min at 300 °C. The total run time was 34 min and 10 s. The transfer line temperature was set to 300 °C and the ion source to 250 °C. The standard electron ionization energy of 70 eV was used to enable the automated identification of metabolites. To avoid filament breakage, the solvent delay of 336 s was selected and applied. Profiles were recorded at 12 spectra/second acquisition rate, in the m/z range from 50 to 635. The general GC/MS suitability, the mass spectrometer accuracy and the presence of leaks in the system were carefully verified within a series of comprehensive autotunes. Quality control solutions consisting of 24 highly pure standards of metabolites (QC), blank samples and biological pools were included in sequence to assess the condition of the system. To acquire and export data in the ANDII MS format, the ChromaTOF software platform for Pegasus BT (ver. 5.32) was applied.

### 2.7. Processing of Obtained Profiles

Collected GC/MS data were exported as ANDII MS and then converted to analysis basis files (ABF) by Reifycs Abf Converter (available at https://www.reifycs.com/AbfConverter). The MS Dial software (ver. 3.96) was chosen to perform comprehensive data processing, alignment, identification and preparation for statistical analysis (RIKEN Center for Sustainable Resource Science, available at http://prime.psc.riken.jp/Metabolomics_Software). Data were analyzed in the range of m/z from 50 to 635, and the retention time range from 6.4 to 25 min. To detect a peak, the minimum height of 55,000 and the mass accuracy of 0.2 Da were required based on the analysis of previously injected retention indices, QCs and biological pools. Features identified by the software as metabolites were kept. Then, peaks were smoothed by the Savitzky–Golay filter method with the smoothing level of 2 scans and the average peak width of 12 scans, respectively. The deconvolution was performed in the next step, applying the EI spectrum cut off of 12 amplitudes and the sigma window value of 0.5 as a compromise between the maximum number of chromatographic peaks resolved and the exclusion of a chromatographic noise. A set of alkane series mixture from C10 to C36 was used in order to determine the retention index for each compound. Metabolites were identified by the library search, i.e., Fiehn with RI as well as with general EI-MS with applied RI, both with 15,302 records (available at http://prime.psc.riken.jp/Metabolomics_Software/MS-DIAL). The following identification settings were chosen: the retention index tolerance of ± 35, the *m/z* tolerance of 0.5 Da, the EI similarity cut off of 60% and the total score cut off of 60% (including the retention time and/or the retention index information in the identification process). A particular analyte identification was considered a match after careful visual evaluation of obtained spectra (vs. proposed library scores) and the verification of calculation by the software similarity indices. Then, data were aligned. The retention time tolerance for the alignment was set to 0.075 min, and the EI similarity tolerance to 60%. Gaps were automatically interpolated by the MS Dial software algorithm, called gap-filling. Features with the sample to blank fold-change ratio lower than 5 were automatically filtered out. Finally, obtained data were normalized to the total ion current of identified metabolites and exported in the normalized form as.txt file. Artifacts, e.g., column bleeds, plasticizers, polysiloxanes, residues of derivatization agents and hydrocarbons, were manually removed before the statistical analysis.

### 2.8. Statistical and Semiquantitative Analysis

Eight samples, representative for each group, were subjected to the statistical analysis. The obtained table of data was imported as a.csv file into the MetaboAnalyst 4.0 platform to perform general statistical analysis and visualization (available at https://www.metaboanalyst.ca) [20]. The k-nearest neighbor method was used to estimate the remaining missing values. No additional filtering procedures were applied. Data were subsequently log-transformed, and the normality was evaluated by the Lilliefors test followed by the visual assessment of q-q plots and the homoscedasticity by Bartlett’s test, respectively (RStudio Version 1.2.5019). Differences in metabolite abundances between three groups were analyzed using the ANOVA test or the Kruskal–Wallis test, with regard to the normality and the homoscedasticity of data. The post hoc Tukey’s HSD test was used to estimate the significance of differences between particular groups when the corrected ANOVA indicated the difference in the signal distribution. For the comparison of the group of rats on a standard diet (no supplementation) and the one supplemented with nanoparticles of zinc, the t-test, the Welch’s t-test and the Wilcoxon rank-sum test were used, depending on the normality and homoscedasticity of data. All p-values were corrected using the Benjamini–Hochberg FDR approach. The principal component analysis (PCA) was conducted for the data overview. As a pretreatment method before the PCA analysis, auto-scaling was used [21]. The heatmaps were prepared for the illustration of relationships between experimental groups with the standard Euclidean distance measure method and Ward’s clustering algorithm, in both cases, respectively.

## 3. Results

### 3.1. Histopathological Examination of Tumors after Treatment with Zn NP

**Figure 2:**
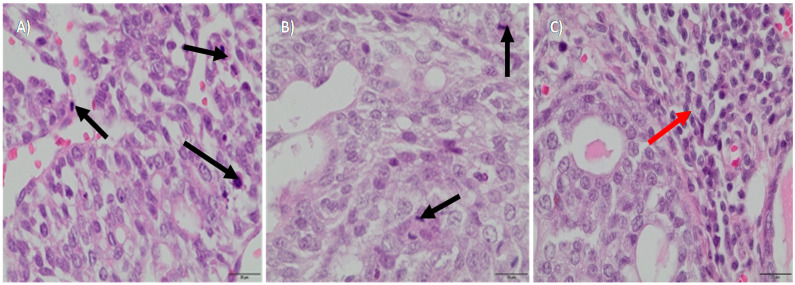
Biopsy images of tumor samples with 40x magnification; hematoxylin and eosin staining was applied. DMBA (7,12-dimethylbenz[a]anthracene) -induced tumor in the control group—II grade adenocarcinoma (**A**), in the group with the zinc microparticles supplementation—II grade adenocarcinoma (**B**), in the group supplemented with zinc nanoparticles—I grade adenocarcinoma (**C**). Black arrows indicate areas of increased proliferation (**A**,**B**). Red arrow indicates inflammatory infiltrates (**C**).

We aimed to determine the potential in vivo cytotoxicity of Zn NP against breast cancer and potential outcomes in the global blood metabolome. In our study, animals were assigned to three dietary groups: all three groups received standard fodder, second and third groups were additionally supplemented with micro- and nanoparticles of zinc, respectively. The supplementation was administered to the rats from 40 days up to 20 weeks of life, at the dose two times exceeding the standard fodder level of zinc. No adverse effects or poor health were observed at the selected dose of supplementation with zinc. As DMBA in rapeseed oil was applied to induce the development of a tumor, its growth was examined by palpation in each group. In the control group (no additional zinc supplementation) and in rats supplemented with zinc microparticles, rapid progression of the disease was observed. No significant differences between these two groups were determined, i.e., several tumors were palpable in both of them. However, the supplementation with nanoparticles of zinc resulted in a strong inhibition of tumor development, as well as in the reduction of the number of palpable tumors at the selected dose. After 150 days of life, the rats were sacrificed, and tumor samples were collected for a histopathological examination (Table 2, Figure 2). Moderately differentiated tumor cells, with areas of increased proliferation, characteristic of grade 2 *adenocarcinoma*, were found both in the control and in the group supplemented with zinc microparticles (Figure 2A,B). In contrast, in the group supplemented with zinc nanoparticles, grade 1 tumors were found, and multiple inflammatory infiltrates observed, with abundant lymphocytes around tumor sites (Figure 2C).

### 3.2. The Characterization of Metabolome Components by GC-TOF MS Approach

In the next step, low-molecular-weight metabolites in blood serum were screened. For the preparation of samples and their analysis, we applied the protocol described previously by Oliver Fiehn in 2016 [19]. The extraction with the ice-cold mixture of acetonitrile, isopropanol and water allowed us to separate mostly polar and semi-polar metabolites from the serum, which are all crucial for cancer metabolism. Further, the derivatization with methoxyamine and MSTFA and the subsequent GC/MS analysis enabled us to determine 158 metabolites, including sugars and their derivatives, sugar alcohols, amino acids and their derivatives, carboxylic acids and their derivatives, lipids and purine derivatives (Figure S2). The examples of identifications after deconvolution are presented in Figure S3A–E. Potential identifications were confirmed based on the calculated retention indices, similarity factors and the visual assessment of obtained EI-MS spectrum vs. suggested by the library match. Finally, GC/MS chromatograms were submitted to bioinformatic analysis with the use of recently released MS Dial software [22]. Consequently, the applied GC/MS approach, followed by data handling with MS Dial software, allowed us to determine crucial components of serum metabolome and to prepare a detailed table for statistical analysis.

### 3.3. Determination of Differences in Profiles of Treated and Diseased Rats

**Figure 3:**
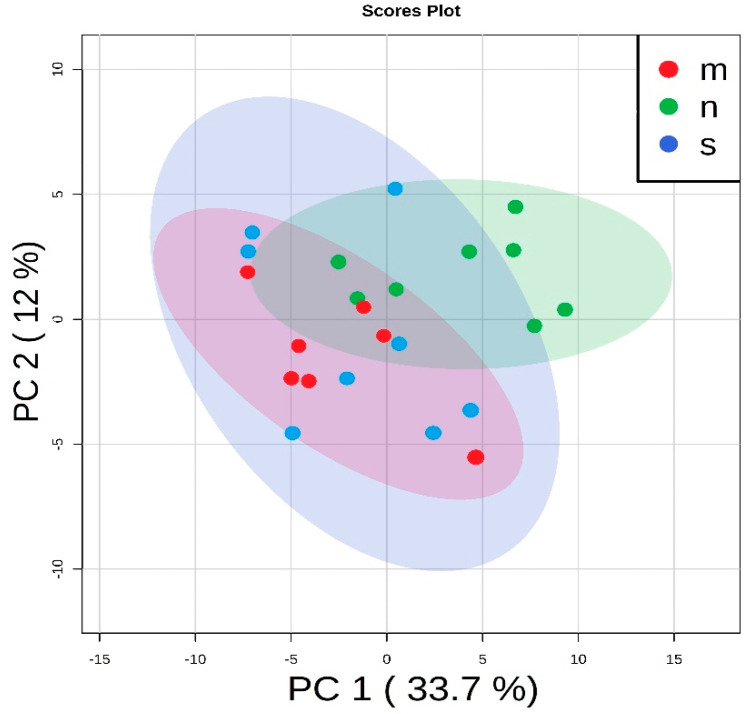
The principal component analysis used for the general data overview: rats on the standard diet (blue dots—s), with the zinc nanoparticles supplementation (green dots—n) and with the zinc microparticles supplementation (red dots—m).

Statistical tests were applied to study differences in abundances of particular metabolites. In the first step, the unsupervised principal component analysis (PCA) was used to analyze overall similarities and differences among the three experimental groups. This analysis showed some discrimination between rats with the Zn NP supplementation and the other groups. The results are presented in Figure 3 and Figure S4.

**Figure 4:**
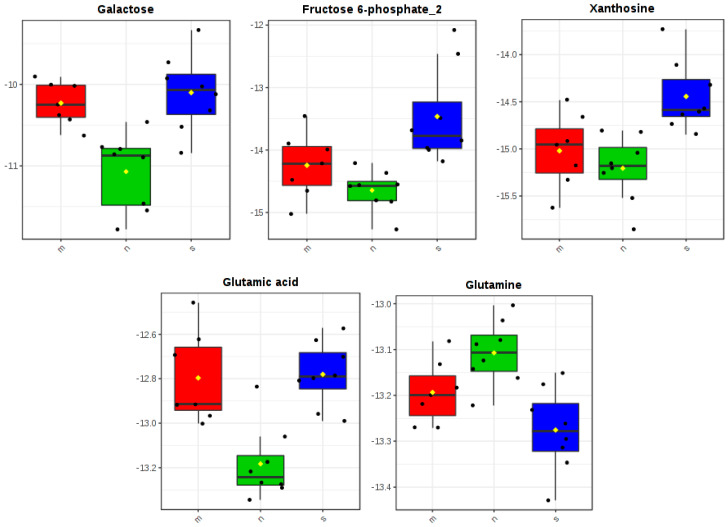
Metabolites that were found to be differentially accumulated across three experimental groups: rats on the standard diet (s), with the zinc nanoparticles supplementation (n) and with the zinc microparticles supplementation (m).

In the one-way ANOVA test, statistically significant differences across the experimental groups were found in abundances of glutamic acid, galactose, glutamine, xanthosine and fructose 6-phosphate (Table S1). Tukey’s HSD was used to test all pairwise differences. We observed that serum levels of glutamic acid, galactose, xanthosine and fructose 6-phosphate were lower in the group supplemented with zinc nanoparticles than in the non-supplemented one. Simultaneously, higher blood serum glutamine was determined in these rats, compared with the non-supplemented group. Rats treated with microparticles of zinc had lower levels of xanthosine and fructose 6-phosphate in blood serum than rats in the non-supplemented group. Finally, rats treated with zinc nanoparticles were found to have lower glutamic acid and galactose levels in their blood serum than rats treated with microparticles. All results are summarized in Table 3, and boxplots were used to depict alterations in abundances of particular metabolites across groups—Figure 4 and Figure S5.

**Figure 5:**
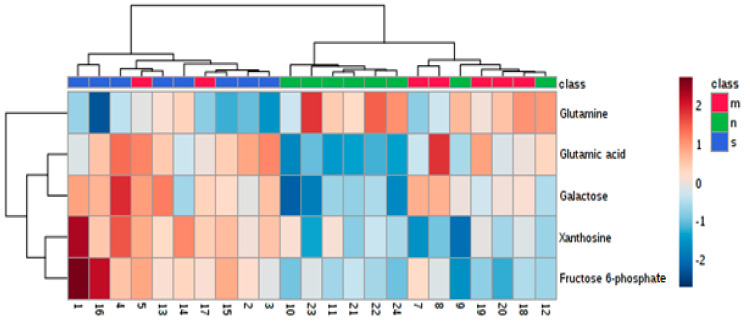
Heatmap for the overview: in both analyses, two separated groups of samples are distinguished, i.e., blue—rats on a standard diet and green—group treated with Zn nanoparticles (NP); inclusions of samples from the third group are observed—labeled in red—group treated with zinc microparticles.

Similar conclusions could be drawn based on the analysis of the generated heatmap (Figure 5). In both analyses, the clear discrimination between the non-supplemented group and those treated with zinc nanoparticles is observed. Furthermore, the group of rats treated with microparticles of zinc indicates similarities to both experimental groups.

**Figure 6:**
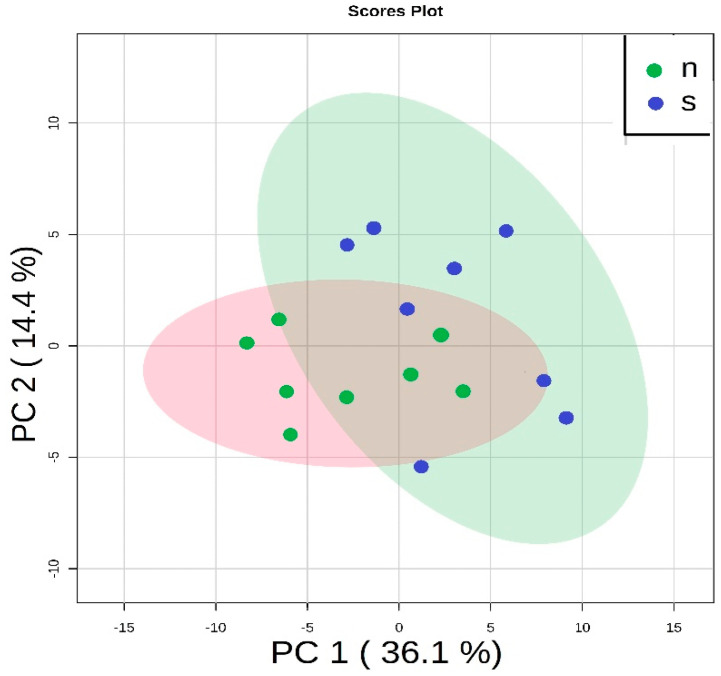
The principal component analysis performed on two groups—rats on the standard diet (blue dots—s) and rats with Zn NP supplementation (green dots—n). Both groups are clearly separated.

Finally, we decided to focus on the comparison of only two groups—the group treated with Zn NP and the non-supplemented group. In this case, the PCA indicated that there were differences between these two groups, and clear discrimination could be observed (Figure 6 and Figure S6).

**Figure 7:**
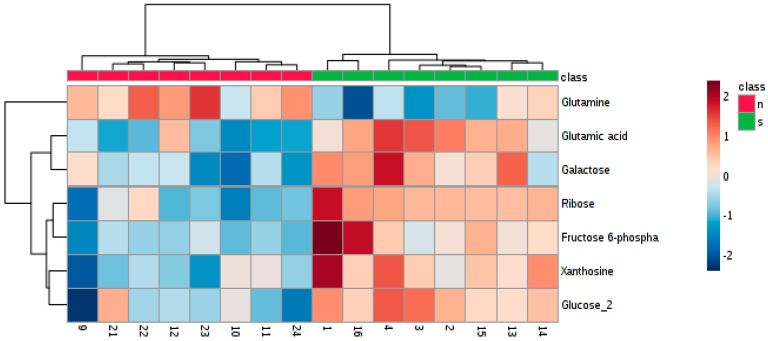
Heatmap for the comparison of two groups: treated with Zn NP (red color—n) and without the treatment method applied (green color—s).

Further, the t-test, the Welch’s test and the Wilcoxon rank-sum test were applied, with regard to the normality of data and the homoscedasticity, respectively (Table S2). The altered abundance of 7 metabolites was found in these tests (FDR corrected *p*-value < 0.05), including ribose, galactose and glucose (sugars), fructose 6-phosphate and xanthosine (sugar derivatives), glutamine and glutamic acid (amino acids). Changes in abundance of all seven metabolites are depicted in Figure S7. The heatmap visualization also showed discrimination between rats fed the standard diet only and those treated with Zn NP (Figure 7). Rats supplemented with Zn NP had lower levels of sugars, their derivates and glutamic acid and a higher level of glutamine in the blood serum compared to non-supplemented rats.

## 4. Discussion

Compared to other inorganic anti-cancer agents, zinc compounds have gained attention owing to their recently promoted safety record. The biological activity and the bioavailability of Zn NP are thought to depend on several factors, for instance, the size, shape, surface charge and purity of obtained nanoparticles. Nanoparticles are able to simply interact with distinct biological molecules and manipulate various cellular cycles, clearly influencing homeostasis [5,6]. In our study, we compared the activity of micro- and nanoparticles of zinc against cancer in vivo. Although we did not measure the exact concentration of zinc in tumor tissues, we hypothesized that zinc nanoparticles of an average size reaching 99 nm could increase the concentration into tumor sites more efficiently, compared to microparticles of an average size of 342 nm. For example, particles of a hydrodynamic diameter less than 100 nm were reported to be the most efficiently delivered in vivo [23]. Therefore, we suspect that nanoparticles of a significantly smaller diameter could enter inside tumor cells more easily, which could thus trigger a biological response more efficiently. On the other hand, the supplementation with zinc microparticles, of lower permeability, had almost no effect on cancer development, as rapid tumor growth was seen during histopathological examination. These findings are consistent with previously published data, which demonstrate no anti-cancer properties in microsized particles of zinc [9].

In turn, our results showed that in rats with DMBA-induced breast cancer, tumor growth was efficiently inhibited, in effect, by the dietary intake of zinc nanoparticles. The therapeutic response to the Zn NP treatment was indicated, for example, by the physical examination, as fewer tumors were palpable in this group of rats. Furthermore, biopsy images of tumor tissues after treatment revealed slower-growing cancer in rats treated with Zn NP. By contrast, in the other groups, multiple areas of increased proliferation of abnormally shaped cells were found, suggesting a rapid development of cancer. Moreover, the infiltration of lymphocytes (tumor-infiltrating lymphocytes—TIL) was recognized in rats treated with Zn NP, based on the microscopic study of biopsies. The presence of TILs may be a favorable prognostic factor in the process of cancer elimination [24]. Rasmussen et al. have already considered the possibility of initiating a natural immune response against cancer in the effect of treatment with Zn NP [4]. Nanoparticles have been found to induce the production of cytokines, of potent anti-cancer properties, for example, the tumor necrosis factor α (TNF-α) [25,26]. Increased production of cytokines has also been reported after the exposure to ZnO NP by inhalation [4,27,28,29,30]. Additionally, cytokines foster the development of cytotoxic cell-mediated immunity, which may enhance the tumor-targeting and inhibition of its growth [4]. Based on our microscopic observations, the in vivo treatment with Zn NP may potentially trigger the activation of a natural immune response against *adenocarcinoma* at the selected dose.

In addition, the determined alterations in serum metabolome of the group treated with Zn NP could also be linked to the inhibition of the development of breast cancer cells. As these cells proliferate intensively, global metabolism usually undergoes extensive reprogramming to facilitate tumor progression [31,32,33]. For instance, excessive generation of low-molecular-weight metabolites becomes a priority, as it may benefit the proliferation of cells. An example is a metabolic switch from oxidative phosphorylation to aerobic glycolysis, i.e., many aggressive cancers avidly consume glucose and produce lactate [32,33]. Although other hexoses like galactose or fructose are less abundant in blood, they can also enter glycolysis under glucose deprivation [32]. High rates of glycolysis ensure the availability of glycolytic intermediates, to enable (i) the synthesis of ribose-5-phosphate and NADPH via pentose phosphate pathway, (ii) the synthesis of glycerol-3-phosphate necessary for the lipid obtainment and (iii) enhanced the biosynthesis of serine and glycine [32]. To maintain required intermediates and glucose levels in the blood, activation of hepatic gluconeogenesis and insulin resistance are considered to be involved [33]. Hence, the decrease in levels of ribose, glucose and galactose, observed in the treated group could be potentially linked to the inhibition of sugar metabolism by cancer cells. Alongside the changes in sugar metabolism, cancer cells have shown high demand for amino acids, most notably glutamine. Glutamine could function as a proteogenic building block, for example, a nitrogen donor, an exchanger for the import of other amino acids, a signaling molecule, etc., with versatile importance to cancer progression [34]. In cancer progression, glutamine is also utilized in the process of gluconeogenesis [32]. We have found a higher amount of glutamine in the Zn NP-treated group, with a simultaneous decrease in glutamic acid level, which suggests a certain reduction in the glutamine demand and metabolism in this group of rats. Xanthosine is also often explored in breast cancer, currently mostly in urine samples [35]. However, the decrease in the abundance of this purine nucleoside in the serum of the rats treated with Zn NP may also indicate the inhibition of the purine metabolism, which is fundamental for cancer cell proliferation [36].

## 5. Conclusions

To conclude, based on our results and recently published in vitro studies, Zn NP could be considered a potential chemotherapeutic agent, inhibiting the progression of breast adenocarcinoma.

## Figures and Tables

**Table 1:** Dynamic light scattering technique (DLS) results for obtained zinc particles.

DLS Parameter	Size (d.nm) ^a^	Z-average (d.nm)	Zeta potential ^b^ (mV)	D ^c^
Zn nanoparticles	99 ± 18	321	+37.1± 7.61	1000
Zn microparticles	342 ± 6.6	3058	+26.7 ± 7.12	1000

^a^—size ± standard deviation; ^b^—zeta potential ± standard deviation; ^c^—dispersity.

**Table 2:** Mammary tumor formation in individual experimental groups treated with 7,12-dimethylbenz[a]anthracene in relation to zinc supplementation

Experimental Group	Number of Tumors in One Rat	Tumor Incidence (%)	Tumor Weight (g)	Tumor Weight Mean (g)	Tumor Grade
Standard diet (no supplementation)	2–9	100%	0.10–7.80	0.89 ± 0.52 *	Adenocarcinoma 2 grade
Supplementation with zinc microparticles	1–6	100%	0.06–7.41	0.68 ± 0.66	Adenocarcinoma 2 grade
Supplementation with zinc nanoparticles	0–3	88%	0.01–1.79	0.40 ± 0.34 *	Adenocarcinoma 1 grade

* *p* = 0.04.

**Table 3:** Metabolites of altered abundance across three groups based on the one-way ANOVA test with results of post hoc tests indicating differences between particular groups.

			Results of Tukey’s HSD Post Hoc Test		
No	Identification	ANOVA Significance FDR Corrected *p*-Value < 0.05	Standard Diet vs. Zinc Nanoparticles Supplementation (S vs. N)	Standard Diet vs. Zinc Microparticles Supplementation (S vs. M)	Zinc Microparticles Supplementation vs. Zinc Nanoparticles Supplementation (M vs. N)
1.	Glutamic acid	0.01497	+		+
2.	Galactose	0.01497	+		+
3.	Glutamine	0.04854	+		
4.	Xanthosine	0.04854	+	+	
6.	Fructose 6-phosphate	0.04869	+	+	

## Supplementary Materials

The following are available online at https://www.mdpi.com/2072-6643/12/11/3457/s1, Figure S1: A schematic representation of the design of the study; Figure S2: Contribution of different classes of metabolites detected in extracts obtained from the rats’ blood serum after the application of protocol proposed by O. Fiehn for the metabolome screening by the GC–MS assay; Figure S3 A–E: Examples of metabolites identified by MS Dial software in serum based on the calculated value of RI and similarity scores. The Fiehn metabolomics library was used for the identification process; Figure S4: The principal component analysis used for the general data overview. Score plots show some discrimination between groups: rats with the zinc nanoparticles supplementation (green dots—n) and the other groups: rats on a standard diet (non-supplemented group) - (blue dots—s) and with the microzinc supplementation (red dots—m); Figure S5: Metabolites which were found to be differentially accumulated across three experimental groups. Blue lines indicate the pairwise differences (FDR < 0.05); Figure S6: the principal component analysis performed on two groups—rats on the standard diet (green dots—s) and rats with Zn NPs supplementation (red dots—n) (A). Features were additionally auto-scaled. Loading plot (B); Figure S7: Metabolites of significantly altered abundance between the non-supplemented group (green color—s) and the one supplemented with zinc nanoparticles (red color—n); Table S1: Metabolites of altered abundance across three groups based on the one-way ANOVA test with the results of the post hoc test indicating differences between particular groups; Table S2: Metabolites of altered abundance across two groups based on the t-test/Welch’s test/Wilcoxon rank-sum test, in regard to normality and homoscedasticity of data, respectively.
