# Supplementary material for: Effect of Zinc Supplementation on the Serum Metabolites Profile at the Early Stage of Breast Cancer in Rats
Source: Nutrients. 2020 Nov 11;12(11):3457. doi: 10.3390/nu12113457 (PMC7696632; doi:10.3390/nu12113457)
Supplement: Supplementary file 1 [file nutrients-12-03457-s001.pdf]

nutrients

MDPI

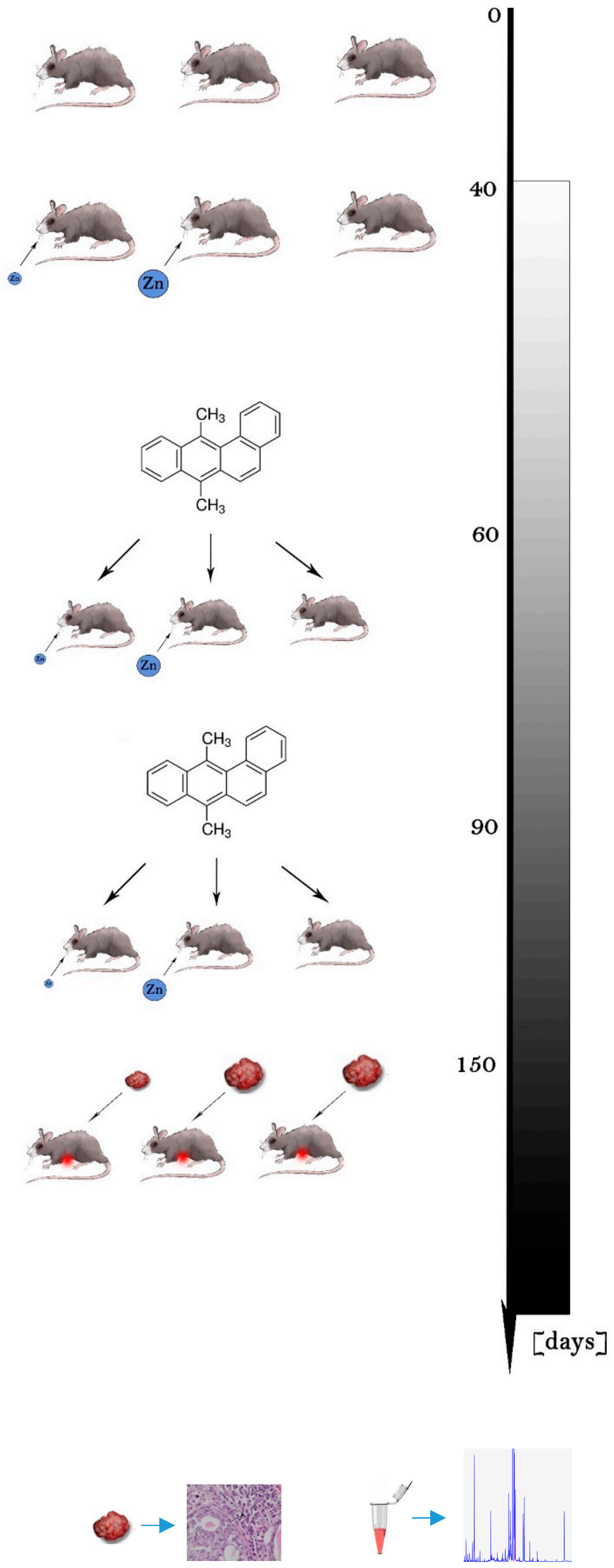

Figure S1. A schematic representation of the design of the study. The supplementation with zinc micro- and nano-particles was started at **40 days** of age and lasted until **150 days of age**. To induce the development of tumor, rats were treated twice with DMBA dissolved in rapeseed was at **60 days of age** and at **90 days of age**, respectively. After **150 days**, the histopathological examination of tumors was performed and the GC-MS profiling of the blood serum metabolome. .

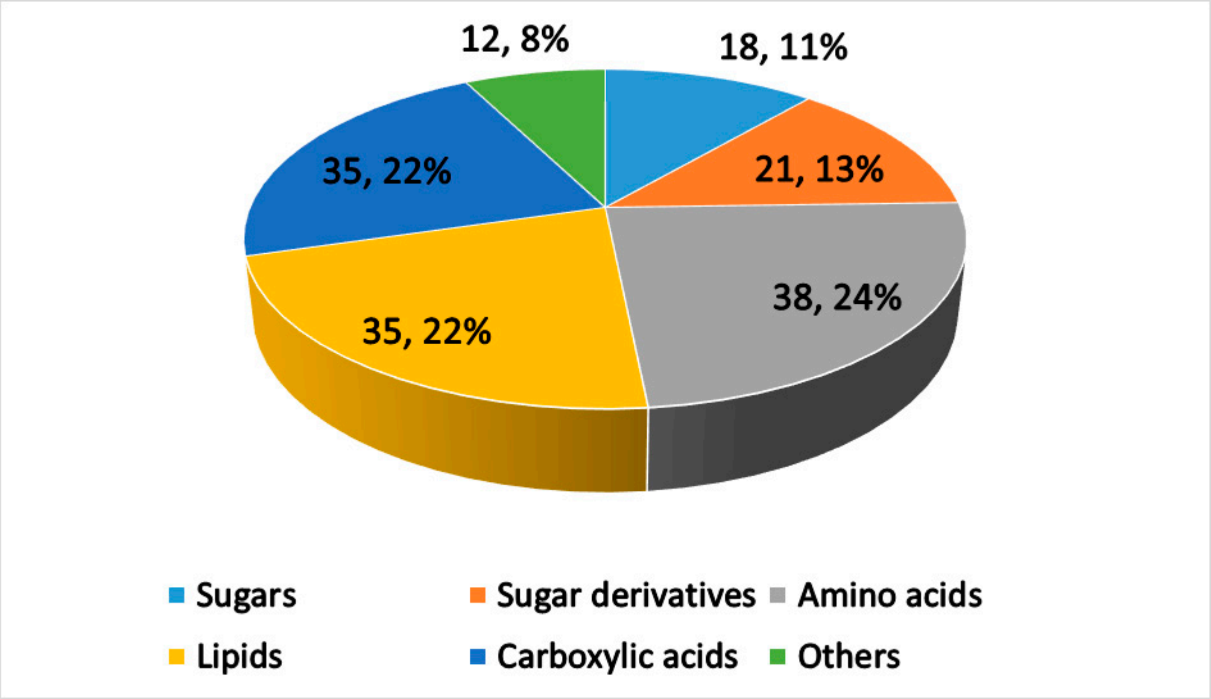

Figure S2. Contribution of different classes of metabolites detected in extracts obtained from blood serum of rats after the application of protocol proposed by O.Fiehn for the metabolome screening by the GC-MS assay.

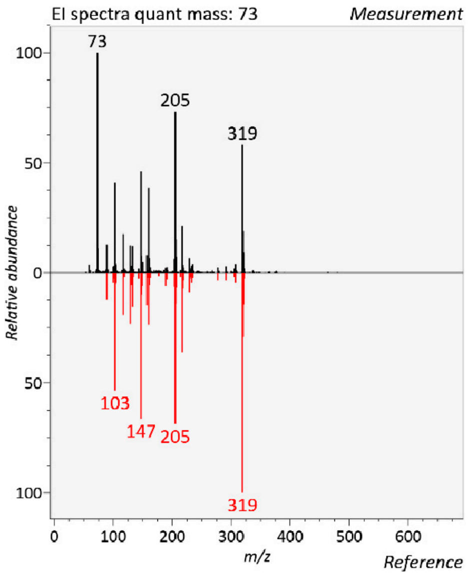

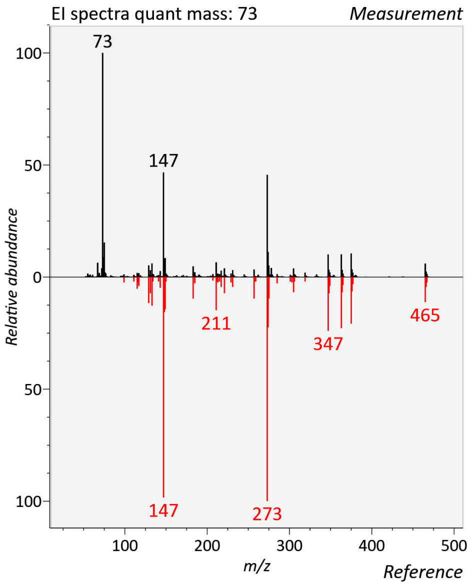

A) Glucose

B) Citric acid

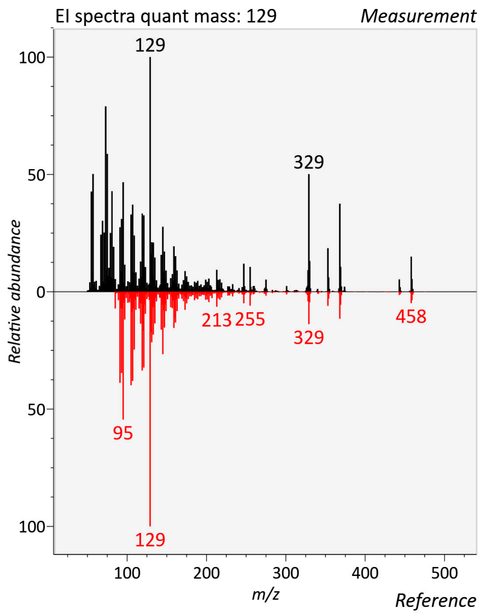

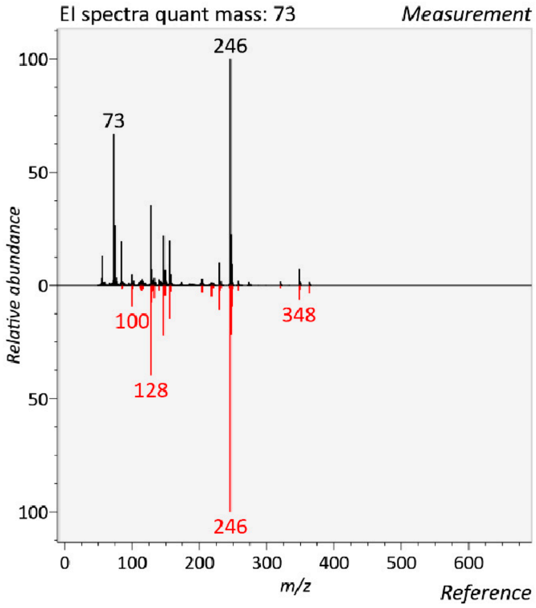

C) Cholesterol

D) Glutamic acid

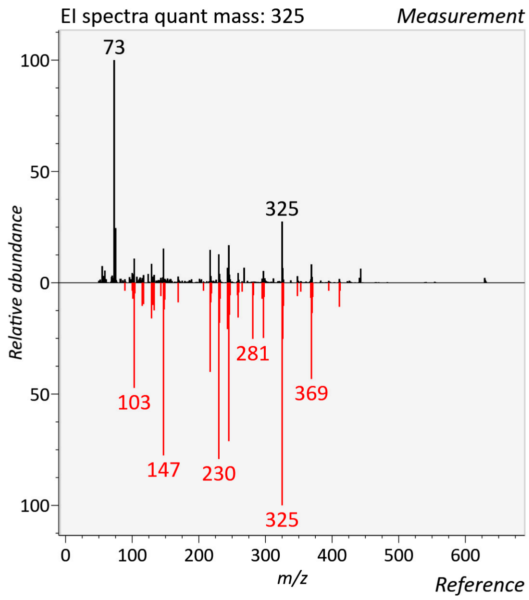

E) Xanthosine

Figure S3. A-E Examples of metabolites identified by MS Dial software in serum based on the calculated value of RI and similarity scores. The Fiehn metabolomics library was used for the identification process.

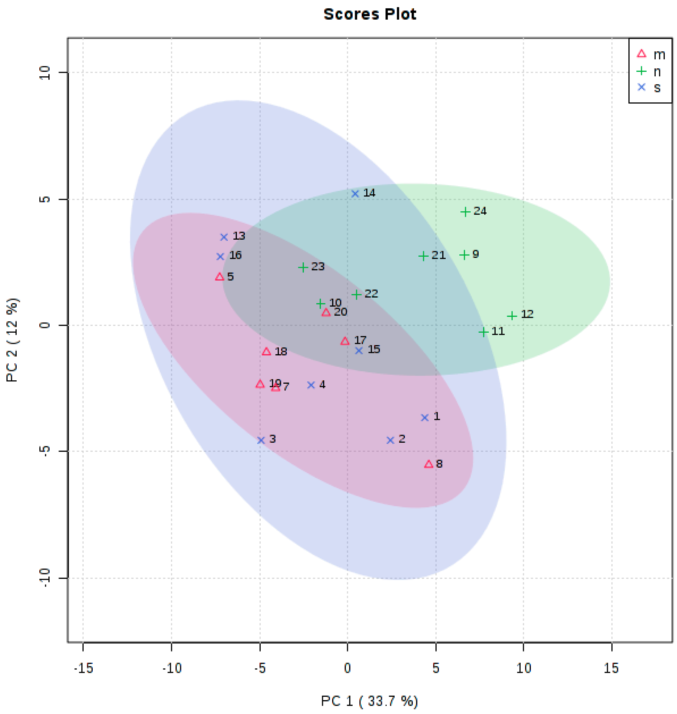
Scores Plot
m
n
s
PC 1 (33.7 %)
PC 2 (12 %)

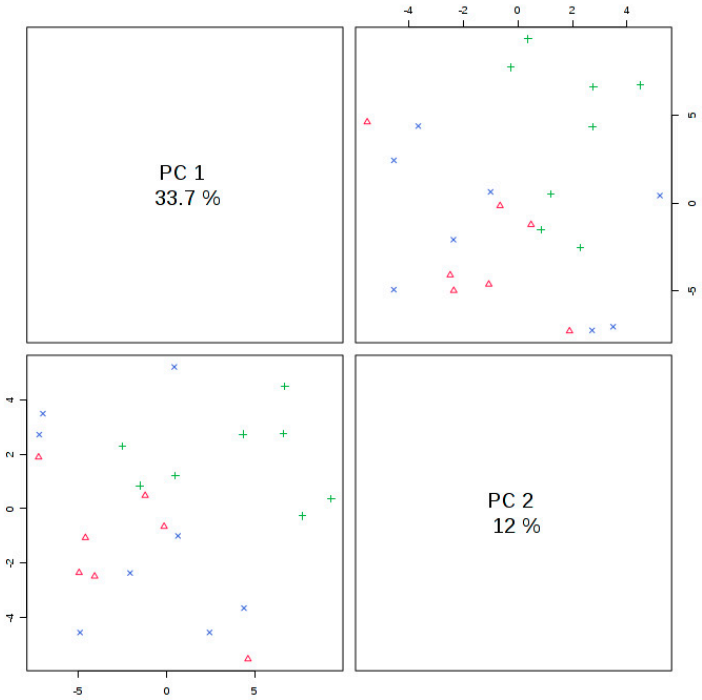
PC 1
33.7 %
PC 2
12 %

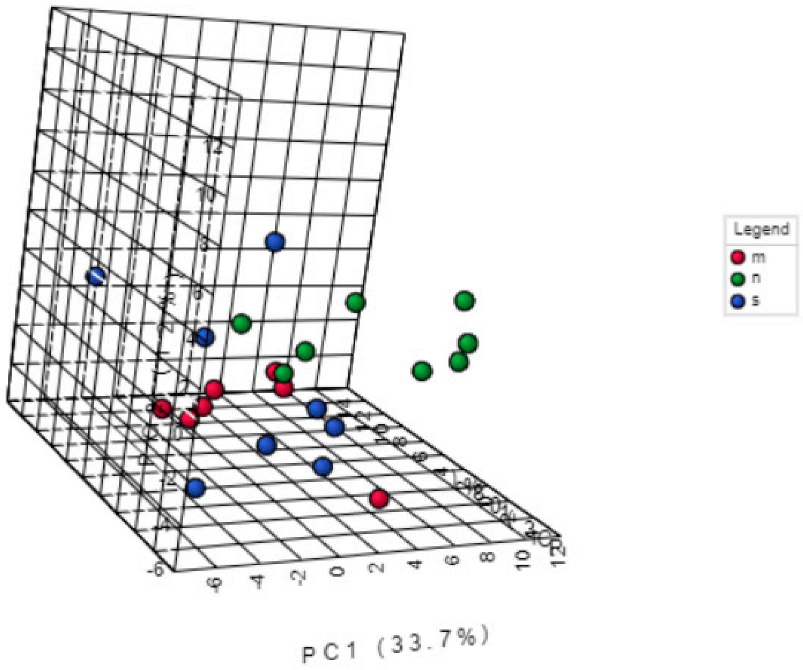

Figure S4. The Principal Component Analysis used for the general data overview. Score plots show some discrimination between groups: rats with the zinc nano-particles supplementation (green dots – n) and the other groups: rats on standard diet (non-supplemented group) - (blue dots – s) and with the microzinc supplementation (red dots – m).

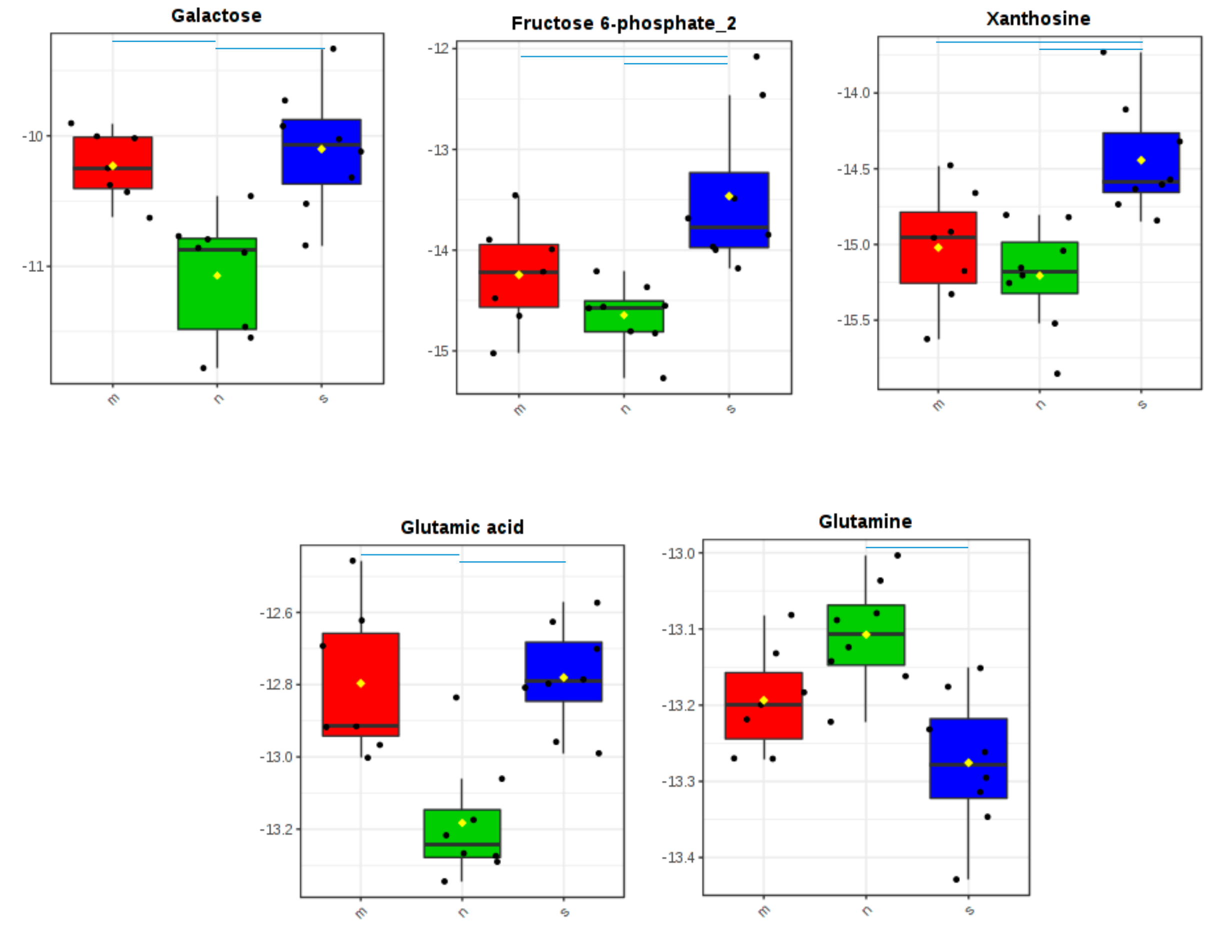

Figure S5. Metabolites which were found to be differentially accumulated across three experimental groups. Blue lines indicate the pairwise differences (FDR < 0.05).

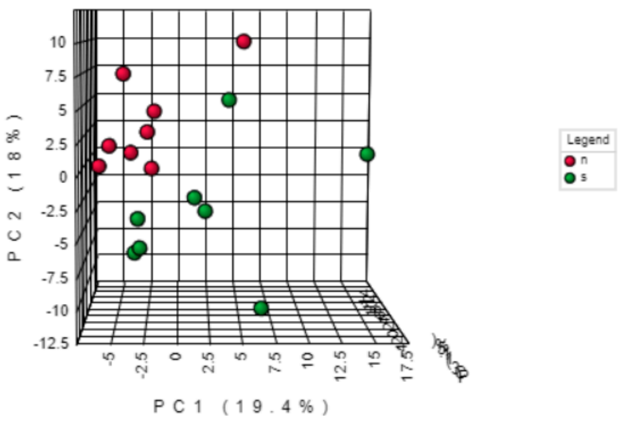

A)

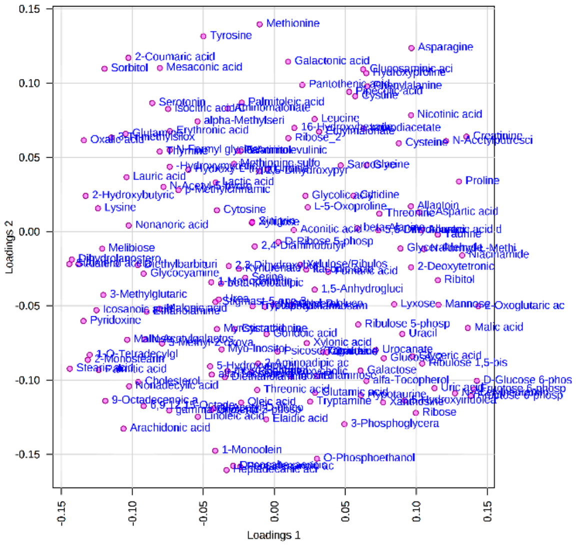

B)

Figure S6. The Principal Component Analysis performed on two groups – rats on the standard diet (green dots - s) and rats with Zn NPs supplementation (red dots – n) (A). Features were additionally auto-scaled. Loading plot (B).

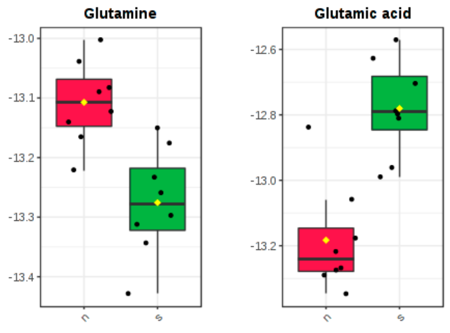

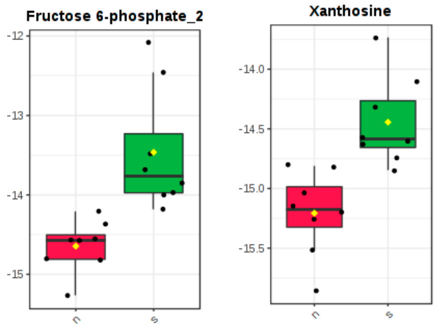

Amino acids **Sugar/Purine derivatives**

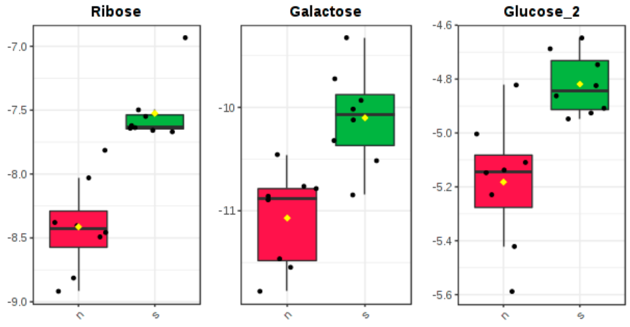

Sugars and sugar alcohols

Figure S7 Metabolites of significantly altered abundance between the non-supplemented group (green color – s) and the supplemented with zinc nanoparticles (red color – n).

Table S1 Metabolites of altered abundance across three groups based on the one-way ANOVA test with the results of the post-hoc test indicating differences between particular groups.

Name	Lilliefors and Bartlett's	-log10(p)	FDR	Post-hoc tests	Remarks
Glutamic acid	**Pos**	3.7431	0.01497	n-m; s-n	-
Ribose	**Neg**	-	-	-	Kruskal Wallis test result negative
Galactose	**Pos**	3.5545	0.01497	n-m; s-n	-
Glutamine	**Pos**	2.8659	0.04854	s-n	-
Xanthosine	**Pos**	2.8217	0.04854	s-m; s-n	-
Fructose 6-phosphate_2	**Pos**	2.7413	0.04869	s-m; s-n	-

Table S2 Metabolites of altered abundance across two groups based on the T-test/Welch's test/Wilcoxon rank sum test, in regard to normality and homoscedasticity of data, respectively.

Name	t.stat/V	*p*-value	-log10(p)	FDR	Remarks
Ribose	0	1.55E-04	3.8085	0.01251	Wilcoxon rank sum
Glutamic acid	-5.1635	1.44E-04	3.842	0.01158	T-test
Xanthosine	-4.2303	8.40E-04	3.0759	0.02673	T-test
Galactose	-4.1657	9.52E-04	3.0214	0.02673	T-test
Glutamine	4.1425	9.96E-04	3.0017	0.02673	T-Test
Fructose 6-phosphate_2	-3.9908	0.00134	2.8729	0.03082	T-Test
Glucose_2	-3.9026	0.00159	2.7976	0.03207	T-Test
Fructose 6-phosphate	-3.6611	0.00257	2.5905	0.04134	T-Test
